# Open questions: how do engineered nanomaterials affect our cells?

**DOI:** 10.1186/s12915-020-00922-0

**Published:** 2020-11-24

**Authors:** Daniela Barrios, Laura Segatori

**Affiliations:** 1 Department of Bioengineering, Rice University, Houston, TX USA; 2 Department of Chemical and Biomolecular Engineering, Rice University, Houston, TX USA; 3 Department of Biosciences, Rice University, Houston, TX USA

**Keywords:** Nanomaterials, Autophagy, Nanotoxicity, Nano-bio interface, Biomimetic nanoparticles

## Abstract

Our cells have evolutionarily conserved mechanisms that battle foreign and toxic materials to maintain cellular homeostasis and viability. How do these cellular machineries respond to engineered nanomaterials?

## How do nanomaterials affect biological systems?

The use of nanomaterials in industrial and biomedical applications has prompted questions about the effects of nanomaterials on living systems, and ultimately human health. A wide range of consumer products ranging from sunscreens to candies contain engineered nanomaterials [1]. Conversely, nanomaterials are also used for biomedical purposes, such as the delivery of drugs, for instance in breast cancer chemotherapeutics [1]. We need, therefore, a detailed understanding of how nanomaterials affect our bodies to engineer safe nanomaterial-based products for human use.

Nanomaterials can be manufactured from a variety of chemical elements through either the controlled assembly of atoms and molecules or the breakdown of larger materials into nano-sized structures. Nanomaterials are generally defined as having at least one dimension that is less than 100 nm and they may be produced in the form of particles, tubes, rods, or fibers. While they may present the same composition as known materials in bulk form, due to their nano-size and high surface area, nanomaterials often possess unique physicochemical properties. Leveraging these unique properties at the nanoscale allows designing nanomaterial-based products with desired and often precisely tunable features for a diverse range of applications.

The features of nanomaterial-based products depend on the specific physicochemical properties of nanomaterials, which can be easily modulated with respect to core composition, size, shape, charge, and surface functionalization. These properties determine the nature of the interaction with cells and organisms and, ultimately, our bodies. For this reason, there is an emerging need to characterize these interactions as a function of nanomaterial physicochemical properties.

Most research efforts have focused on toxicology studies as part of the safety analysis of nanomaterials used in biomedical applications. Even nanomaterials that are considered safe based on cell death studies are likely to affect biological systems. However, the interactions between nanomaterials and human cells and their components remain largely uncharacterized, and fundamental questions about cellular responses ranging from the uptake of nanomaterials, to their effects on specific cellular machineries, are only now being explored. As engineered and natural nanomaterials share common properties, studies of cellular interactions with naturally occurring nanosized organisms and materials such as microorganisms, protein aggregates, and toxins provide a valuable source of information for addressing questions on the effects of engineered nanomaterials on human cells.

## How do cells interface with nanomaterials?

Upon localization onto the cell membrane, engineered nanomaterials are internalized through innate endocytic pathways, which also mediate uptake of bacteria and viruses. Nanomaterials may engage different endocytic pathways ranging from pinocytosis of nanomaterials in extracellular fluid to phagocytosis of nanoparticles coated with immunoglobulin and complement proteins [2]. The uptake mechanism generally depends on the nanomaterial physicochemical properties, such as size, charge, and surface functionalization. Gold nanoparticles, for instance, present great potential as drug delivery carriers and have been modified at their surface to achieve the desired uptake properties; surface modifications resulting in a positively charged nanoparticle surface typically lead to enhanced cellular uptake compared to neutral or negatively charged gold nanoparticles, while coating of the nanomaterial with polyethylene glycol decreased serum protein adsorption and phagocytosis [2]. It is often difficult to establish the nanomaterial uptake mechanism, however, as multiple mechanisms may be engaged simultaneously, especially at high nanomaterial concentrations.

The biological identity of nanomaterials also seems to play an important role in cellular uptake. The biological identity is largely dictated by the protein corona, a protein coat on the surface of nanomaterials that forms through the adsorption of proteins from the blood or cell culture media. As the protein corona is the first to interface with the cell membrane, the biological identity can greatly impact the cellular uptake of nanomaterials [3].

Predicting the combinatorial effect of these multiple factors on cellular uptake remains a challenge. Characterization of the critical role of the protein corona, however, has motivated the development of new strategies that focus on modulating the surface of nanomaterials and tuning the protein corona to obtain a more predictable uptake response [3].

## How do nanomaterials interact with the intracellular machinery?

Upon internalization through pathways commonly used by invading pathogens, nanomaterials are likely to be perceived by cells as foreign and toxic. Not surprisingly, they immediately become participants in a battlefield where host cells direct and manage a clearance apparatus specifically tailored to invading pathogens. This cellular response is actuated by the autophagy-lysosome system, a coordinated machinery that mediates the degradation of a diverse range of nano-sized materials including protein aggregates, damaged organelles, and foreign entities [4, 5]. Similar to the battle against invading pathogens, which may result in pathogen neutralization or invasion, the battlefield between autophagy and nanomaterials also results in a bifunctional interaction precisely shaped by the nature of the nanomaterial physicochemical properties. Since autophagy serves as the first line of defense against nanomaterials, any effect of nanomaterials on cellular components and systems occurs after nanomaterials are processed by the autophagy-lysosome system. The interaction of nanomaterials with autophagy also shapes the fate of nanomaterials inside cells. Understanding what happens at this interface is, therefore, critical for the design of nanomaterials with predictable functionalities.

## How does the autophagy pathway interact with nanomaterials?

**Fig. 1.**
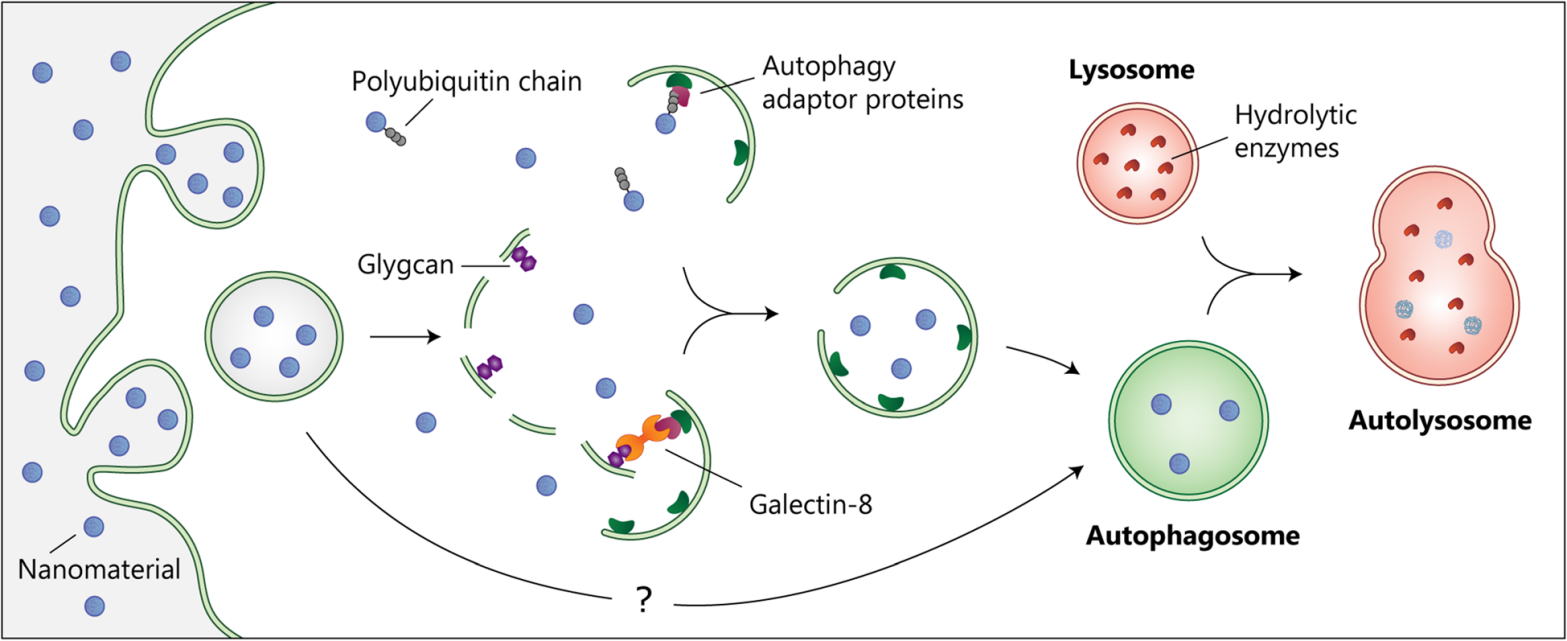
Autophagic response to engineered nanomaterials. Upon internalization, nanomaterials may escape the endocytic vesicle through disruptions of the endosomal membrane. Once in the cytoplasm, nanomaterials can be recognized by autophagy adaptors proteins or danger receptors that recruit isolation membranes. Elongation of the isolation membranes sequesters nanomaterials inside vesicles called autophagosomes, which fuse with lysosomes to form autolysosomes. Autolysosome formation may be followed by enzymatic degradation or secretion. The mechanism of autophagy activation in response to nanomaterials that remain within the endocytic pathway remains unclear

Autophagy is an evolutionarily conserved process that proceeds through the sequestration of cargo material inside double-membrane vesicles in preparation for degradation. Natural as well as engineered nanomaterials are often tagged with “eat-me” signals such as poly-ubiquitin chains which are recognized by autophagy adaptor proteins and targeted to isolation membranes (Fig. 1) [4]. Elongation of the isolation membranes results in the formation of autophagosomes that entrap the cargo material. Fusion of autophagosomes with lysosomes leads to the formation of autolysosomes, which, in turn, results in cargo degradation by hydrolytic enzymes or secretion via exocytosis [5].

While some nanomaterials can enter the cytoplasm, internalized nanomaterials are mainly localized in endocytic vesicles. It is unclear, however, how nanomaterials inside vesicles are recognized by the autophagy pathway. Some nanoparticles are capable of endosomal escape through mechanisms such as the “proton sponge” effect, which ruptures the endosomal membrane [6]. Rupture of the endosomal membrane is likely to be recognized by the autophagy system through the same mechanism that recognizes bacteria-induced membrane disruption. Invasion of *Salmonella typhimurium*, for instance, proceeds through the disruption of enclosing double-membrane vesicles and exposure of complex β-galactoside-containing glycans to the cytoplasm [4]. Because the cytoplasm is free of complex sugars under physiological conditions, the glycans from the inner membrane of the damaged vesicles are rapidly recognized by the danger receptor galectin-8 that mediates the recruitment of the autophagy machinery. Nanomaterials that reach the cytosol through membrane disruptions will likely also be recognized by galectin-8 and promote an autophagic response (Fig. 1) [7].

The mechanisms underlying the interaction of the autophagy pathway with endocytosed nanomaterials that cannot escape into the cytosol remains poorly characterized. Evidence of crosstalk between the endocytic and autophagy pathways supports the notion of cargo movement between these systems [8]. While advanced imaging methods, such as confocal microscopy, have been critical in documenting cellular trafficking from endocytic vesicles to autophagosomes [8], we still lack a detailed understanding of the signaling cues that govern cargo movement between these pathways.

## How do nanomaterials affect cellular processes associated with autophagy?

Internalization of nanomaterials, which are likely perceived as foreign or toxic nanosized structures, results in the activation of autophagy in an attempt to enhance the cellular clearance capacity. Nanomaterials, however, can also impair the autophagy pathway. The impairment of autophagic clearance observed upon nanomaterial uptake is typically due to interactions between nanomaterials and specific components of the autophagy system, resulting in the dysfunction of critical steps of this degradation pathway. Evidence of excessive autophagy activation has been correlated to nanomaterial toxicity, but a direct causal relationship between autophagy activation and cell death is still lacking.

Autophagy impairment typically manifests as an increase in autophagosome formation that is not followed by enhanced degradation, suggesting a blockage in the autophagic flux. Impairment of the autophagy pathway has been documented upon cellular uptake of diverse nanomaterials, such as gold, iron oxide, and silica nanoparticles [5]. The mechanistic details of nanomaterial-induced autophagy impairment are not known, but multiple studies point to the disruption of autolysosome formation and lysosomal function as the underlying causes.

The cytoskeleton plays an important role in the trafficking of vesicles inside cells, supporting the formation of autophagosomes and the fusion of autophagosomes to lysosomes. Impairment of cytoskeleton function can thus result in disruption of autolysosome formation. Nanomaterials such as titanium dioxide and gold nanoparticles can cause damage to the cytoskeleton, leading to autophagy dysfunction [5]. Possible mechanisms of nanomaterial-induced damage of the cytoskeleton are inhibition of microtubule polymerization, disruption of actin networks, and production of reactive oxygen species leading to microtubule instability and fracture of actin filaments [5, 9].

Proper lysosomal function is also necessary for the progression of the autophagic flux. Lysosomal membrane permeability and inhibition of lysosomal enzymes can greatly disrupt lysosome function and fusion to autophagosomes. Several nanomaterials have been reported to disrupt lysosomal function by causing acidification of the lysosome due to the large buffering capacity of cationic species [5]. Cationic polystyrene nanoparticles were found to induce lysosomal permeability, reduce the formation of autolysosomes, and increase cytotoxicity compared to neutral or anionic polystyrene nanoparticles [6]. Zinc oxide nanoparticles, which produce cations upon dissolution, also induced lysosomal instability and decreased viability [5]. Lysosomal dysfunction, therefore, does not only cause autophagy impairment but can also lead to cell death.

Despite the strong correlation between autophagy and cell death upon the uptake of some nanomaterials, the detailed role of autophagy in nanotoxicity remains unknown. Whether autophagy impairment directly functions as a pro-death signal or whether the crosstalk between apoptosis and autophagy promotes programmed cell death remains subject of intense debate. Understanding the mechanism of cell death upon nanomaterial internalization is thus crucial for engineering safe nanomaterials.

## How can we modulate the interactions of nanomaterials and cells?

The field of nanotechnology is using biomimetic strategies informed by cell biological research to design nanoparticles with predictable functionalities for biomedical applications. For instance, years of extensive research on the molecular mechanisms governing double-membrane vesicle formation, pathogen invasion, and intracellular protein aggregation provide foundational knowledge on the way nanomaterials interface with cellular components and systems.

Nanomaterials coated with naturally occurring biomolecules that have evolved to mediate specific biological features allow controlled modulation of cellular interactions. For instance, cell-penetrating peptides mediating virus penetration through the cell membrane have been used to increase cellular uptake of nanomaterials [2]. Similarly, surface functionalization of nanomaterials with signaling molecules that bind specifically to cell surface receptors enables targeting of nanomaterials to a desired cell type [2]. Cell membrane coating of nanoparticles can further preserve the complexity of the outer layer of cells and transfer natural biointerfacing capabilities to a variety of nanomaterials, enhancing nanomaterial specificity and safety [10]. Our understanding of the autophagic response to natural nanosized structures such as aggregated proteins and organelles can be also leveraged to design nanomaterials with desired effects on the autophagy system. Such strategy provides an interesting approach to develop nanotherapeutics for the treatment of diseases characterized by inefficient autophagic clearance and accumulation of storage material as well as for the design of delivery systems that evade autophagy and enhance drug delivery.

Biomimetic strategies can greatly aid the design of safe nanomaterials for human use while the major open questions concerning the interactions between nanomaterials and human cells remain under investigation. By harnessing the properties of well-characterized naturally occurring nanosized materials, we can more effectively predict the bifunctional interaction between biomimetic nanomaterials and cells and thus design safe nanomaterial-based products for industrial and biomedical applications.

## Data Availability

Not applicable.
